# The Porcine MicroRNA Transcriptome Response to Transmissible Gastroenteritis Virus Infection

**DOI:** 10.1371/journal.pone.0120377

**Published:** 2015-03-17

**Authors:** Xiao Liu, Ling Zhu, Shan Liao, Zhiwen Xu, Yuancheng Zhou

**Affiliations:** 1 Animal Biotechnology Center, College of Veterinary Medicine, Sichuan Agricultural University, Ya’ an, China; 2 Key Laboratory of Animal Disease and Human Health, College of Veterinary Medicine, Sichuan Agricultural University, Ya’ an, China; 3 Liver Center and Gastrointestinal Division, Department of Medicine, Massachusetts General Hospital, Harvard Medical School, Boston, Massachusetts, United States of America; Institut Pasteur of Shanghai, Chinese Academy of Sciences, CHINA

## Abstract

Transmissible gastroenteritis virus (TGEV; *Coronaviridae* family) causes huge economic losses to the swine industry. MicroRNAs (miRNAs) play a regulatory role in viral infection and may be involved in the mammalian immune response. Here, we report a comprehensive analysis of host miRNA expression in TGEV-infected swine testis (ST) cells. Deep sequencing generated 3,704,353 and 2,763,665 reads from uninfected ST cells and infected ST cells, respectively. The reads were aligned to known *Sus scrofa* pre-miRNAs in miRBase 19, identifying 284 annotated miRNAs. Certain miRNAs were differentially regulated during TGEV infection. 59 unique miRNAs displayed significant differentially expression between the normal and TGEV-infected ST cell samples: 15 miRNAs were significantly up-regulated and 44 were significantly down-regulated. Stem-loop RT-PCR was carried out to determine the expression levels of specific miRNAs in the two samples, and the results were consistent with those of sequencing. Gene ontology enrichment analysis of host target genes demonstrated that the differentially expressed miRNAs are involved in regulatory networks, including cellular process, metabolic process, immune system process. This is the first report of the identification of ST cell miRNAs and the comprehensive analysis of the miRNA regulatory mechanism during TGEV infection, which revealed the miRNA molecular regulatory mechanisms for the viral infection, expression of viral genes and the expression of immune-related genes. The results presented here will aid research on the prevention and treatment of viral diseases.

## Introduction

Transmissible gastroenteritis virus (TGEV), belonging to the *Coronaviridae* family, is the pathogen of transmissible gastroenteritis of swine (TGE). All ages and strains of pigs are susceptible to the virus. Mortality is more than 10% in 2–3-week-old infected pigs, but it is 100% for pigs less than 2 weeks old. Infected animals show clinical symptoms of watery diarrhea, dehydration and vomiting [1].

TGEV is highly contagious and can spread through various channels; however, the main infection route this virus is fecal-oral. At present, the virus is distributed worldwide; the prevalence of TGEV in different regions of China has long been reported, and TGEV infection has caused huge economic losses to the swine industry.

The TGEV nucleic acid is single-stranded RNA with a virus particle diameter of 90–200 nm. The virus particles comprise three major structural proteins: the phosphoprotein (N protein or nucleoprotein) that wraps the virus genome RNA; the membrane-bound protein (M protein or E1 protein), which is embedded in the lipid membrane; and the spike glycoprotein (S protein), which forms the spike on the surface of the virus. The spike glycoprotein may determine the cell tropism and the membrane fusion function of the virus, and it enables the transfer of the virus nucleoprotein into the cytoplasm. The spike glycoprotein also carries major B lymphocyte epitopes, and plays a key role in improving immunity [2, 3].

MicroRNAs (miRNAs) are endogenous, non-coding RNAs, first found in eukaryotes with a length of 20–25 bp. MiRNAs are initially transcribed by RNA polymerase II in the nucleus into large transcripts called primary miRNAs (pri-miRNAs). Primary miRNAs are processed by RNase III enzyme Drosha and the cofactor Pasha into 60 nt–80 nt hairpin structures called precursor miRNAs (pre-miRNAs). Subsequently, precursor miRNAs are transported to the cytoplasm and further cleaved by RNase III enzyme Dicer into 20–25 nt miRNA:miRNA* double strands. One strand of the miRNA duplex is incorporated into the RNA-induced silencing complex (RISC), where the mature single-stranded miRNA plays a regulatory role to target mRNAs [4–8].

MiRNAs play a key role in the regulation of the eukaryotic metabolic processes, including body development, hematopoiesis, organogenesis, cell differentiation, proliferation, apoptosis, fat metabolism, the occurrence of cancer and many other cellular processes [9–11]. MiRNAs can also regulate plant hormone levels and signal transduction. Recent studies indicate that numerous cellular miRNAs play a regulatory role in the interaction network between the host and viruses. The host-encoded miRNAs can regulate the process of viral infection by targeting the viral genome or host genome; e.g., host-encoded miRNAs can promote or inhibit DNA replication of the virus [12]. Recently, the miRNAs regulation in of the immune response by miRNAs has been observed. Evidence suggested that miRNAs are also involved in the mammalian immune response, and play an important role in antiviral processes.

In addition to the cellular miRNAs from eukaryotes, recent research also confirmed the existence of viral-encoded miRNAs, which mediated the silencing of host genes by targeting the host mRNAs. Viruses could evade recognition and destruction by the host immune system through this approach, such that the host acquires a long-term latent infection with the virus [13]. In addition, the virus-encoded miRNAs could also change the infection status of the virus by regulating viral gene expression [14, 15].

MiRNA sequencing techniques based on the Illumina/Solexa high-throughput sequencing platform have overcome the limitations on miRNA research techniques, helping researchers to sequence the specific sized miRNAs from samples directly, to determine miRNA expression profiles and to discover or identify novel miRNAs in organisms without any sequence information. Northern-blotting is the most common method for confirming the expression of miRNAs, but it suffers from low sensitivity and low throughput. By contrast, stem-loop RT-PCR is a sensitive and efficient method for the detection of miRNA that has been widely used in miRNA research [16–18].

To determine the miRNA expression profile of the swine testis (ST) cell line after infection with TGEV, and to screen for miRNAs that play regulatory roles in the process of viral infection, small RNAs from infected and uninfected ST cells (control) were sequenced by a high-throughput sequencing system, and the transcriptome and miRNA expression profiles of both cell types were comprehensively analyzed. This study carried out an in-depth data analysis to identify known and novel miRNAs and other small RNAs based on an miRNA database. We also analyzed the viral and host target genes of the miRNAs with the aim of identifying the functions of these miRNAs.

The results of this research should help in the development of new control strategies to treat or prevent TGEV infection.

## Results

### Overview of the Solexa high-throughput sequencing data

**Fig 1:**
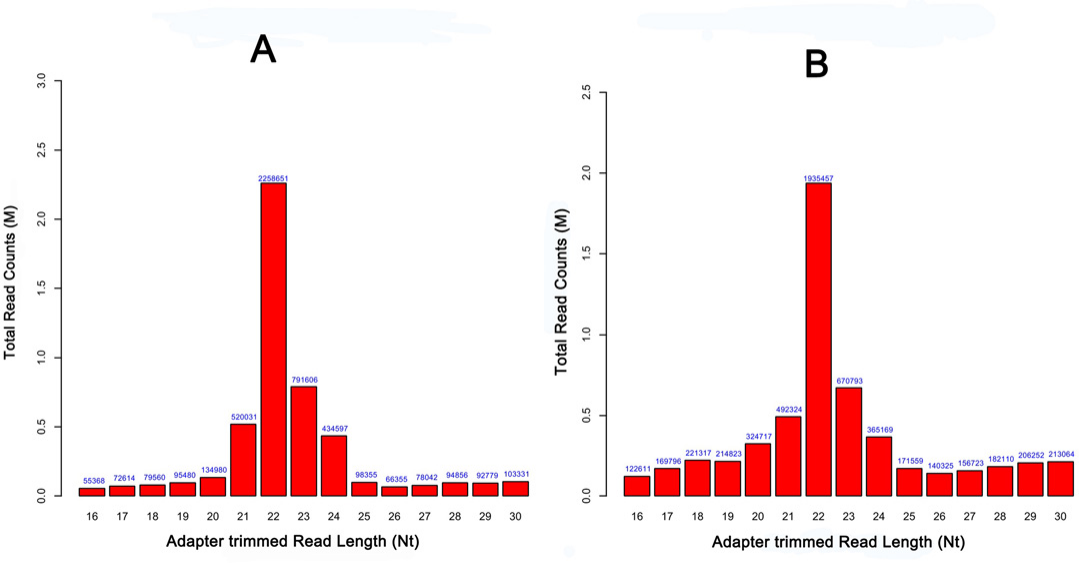
Length distribution of total sRNAs in TGEV-infected ST cells and normal ST cells. (A) Bar chart showing the total read counts against the read lengths for the complete adapter-trimmed read set in TGEV-infected ST cells. (B) Bar chart shows the total read counts against the read lengths for the complete adapter-trimmed read set in normal ST cells. The results indicate a successful enrichment of mature miRNAs in the TGEV-infected ST cells and normal ST cell libraries.

**Fig 2:**
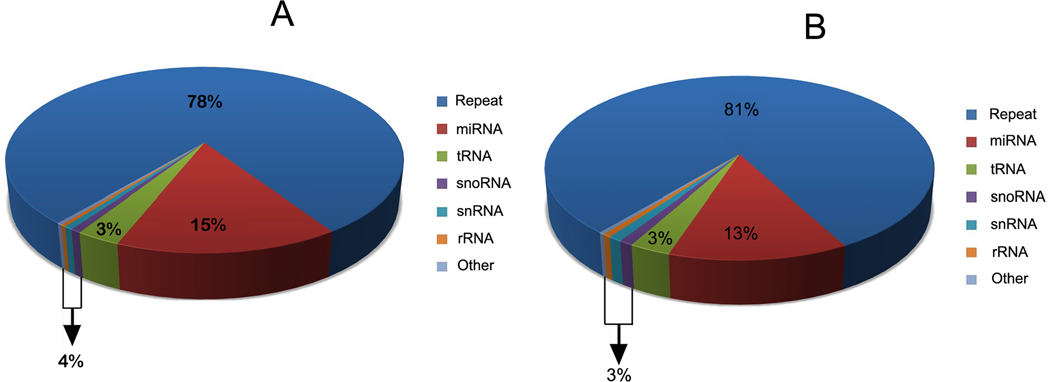
Pie charts of sRNA percentages. (A) Pie chart summarizing the different classes of sRNAs in TGEV-infected ST cells. (B) Pie chart summarizing the different classes of sRNAs in normal ST cells.

To investigate the miRNA expression profiles of infected ST cells, ST cells were infected with TGEV SC strain and the normal ST cell line was used as a control. 15–25 nt sRNAs were isolated and analyzed by deep sequencing. The normal ST cell sample generated 6,150,141 clean reads (reads that pass quality filtering) and 5,877,912 adapter-trimmed reads (reads that have passed quality filtering, adapter filtering and length filtering, length ≥15 nt). The infected ST cell sample generated 7,385,050 clean reads and 6,974,168 adapter-trimmed reads. 3,704,353 and 2,763,665 reads from the ST cell sample and the infected ST cell sample could be aligned to known Sus scrofa pre-miRNAs in miRBase 19, respectively (Table 1) (NCBI GEO Accession number: GSE64737). The length distribution of the adapter-trimmed reads was similar in infected ST and normal ST sample libraries (Fig. 1), and most of the adapter-trimmed reads were 22 nt in length. Pie charts summarizing the different classes of sRNAs in the samples are shown in Fig. 2.

**Table 1:** Overview of miRNA-seq data processing of all samples (single read libraries).

Sample Name	Clean Reads	Adapter-trimmed Reads (length > = 15nt)	Reads aligned to known *Sus scrofa* pre-miRNA in miRBase 19
**Normal ST cell**	6,150,141	5,877,912	3,704,353
**Infected ST cell**	7,385,050	6,974,168	2,763,665

Indicated from left to right are the numbers of reads that passed quality filtering (clean reads), the numbers of reads that passed both quality filtering, adapter filtering and length filtering (Adapter-trimmed reads ≥ 15 nt), and the number of reads that could be aligned to the known *Sus scrofa* pre-miRNAs in miRBase 19 with zero or one mismatch, respectively.

### Analysis of host-encoded miRNA expression profile

In our study, the sequencing data were processed through Illumina’s Genome Analyzer Pipeline, and alignment to the reference genome of *Sus scrofa* identified 525 miRNAs from 477 pre-miRNAs, of which 284 mature miRNAs were annotated in miRBase 19 (S1 Table) and 241 were novel host-encoded miRNAs (S2 Table).

### Differential expressed miRNAs between infected ST cells and uninfected ST cells

The high-throughput sequencing not only identified a series of novel miRNAs, but also provided data about their expression levels. The miRNA expression profile database showed that most miRNAs were expressed by a small portion of miRNA genes. In the miRNA expression profile of the infected ST cell line sample, the expression of 4% (20/491) of the miRNAs accounted for 68.6% of the total expression; and in the miRNA expression profile of the normal ST cell line sample, the expression of 4% (20/500) miRNAs accounted for 73.6% of the total expression. Interestingly, although there were some changes in their ranking, the miRNAs with the highest expression levels were consistent in both samples. Among them, ssc-let-7f, which is derived from the let-7-family, and the ssc-miR-21 had the highest expression level, and this result is consistent with previous studies [19].

Among the 525 sequenced mature miRNAs, 466 (88.8%) unique miRNAs were co-expressed in normal ST cell sample and infected ST cell sample; however, 34 (6.5%) and 25 (4.8%) were preferentially expressed in the ST cell sample and infected ST cell sample, respectively. Among the 241 novel miRNAs, 18 miRNAs were preferentially expressed in the TGEV-infected ST sample, and chr3–10158, chr5–12080, chr5–12157 and chr8–14894 had the maximum expression levels.

**Fig 3:**
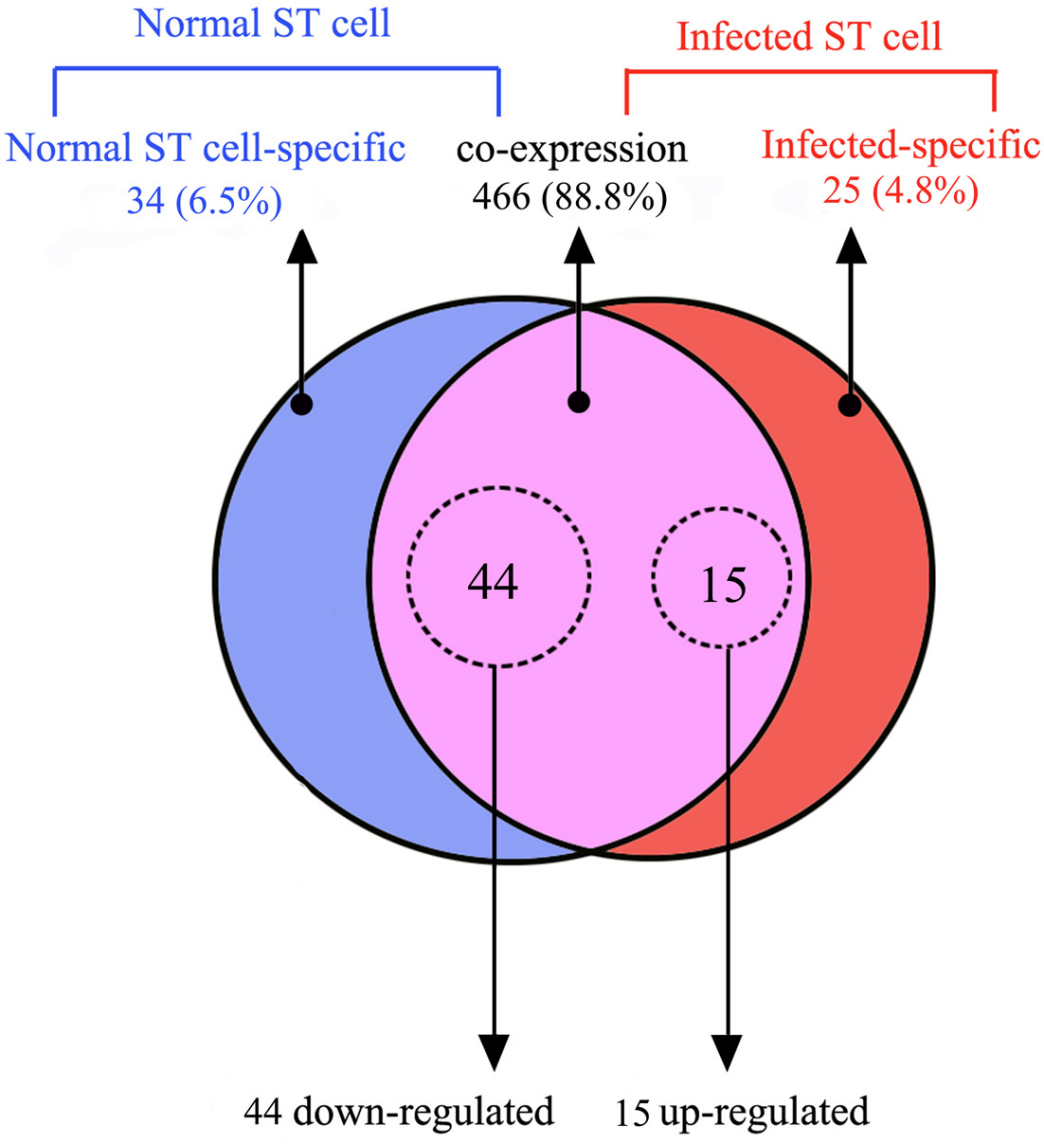
Comparison of differentially expressed miRNAs between the normal ST cell and TGEV-infected ST cell samples (*p*<0.0001). The Venn diagram shows the distribution of 525 unique miRNAs between normal ST cell (left, blue circle) and TGEV-infected ST cell sample (right, red circle) libraries. The overlapping section represents 466 co-expressed miRNAs. The dashed circles indicated the miRNAs that were significantly differentially expressed (*p*<0.0001).

**Fig 4:**
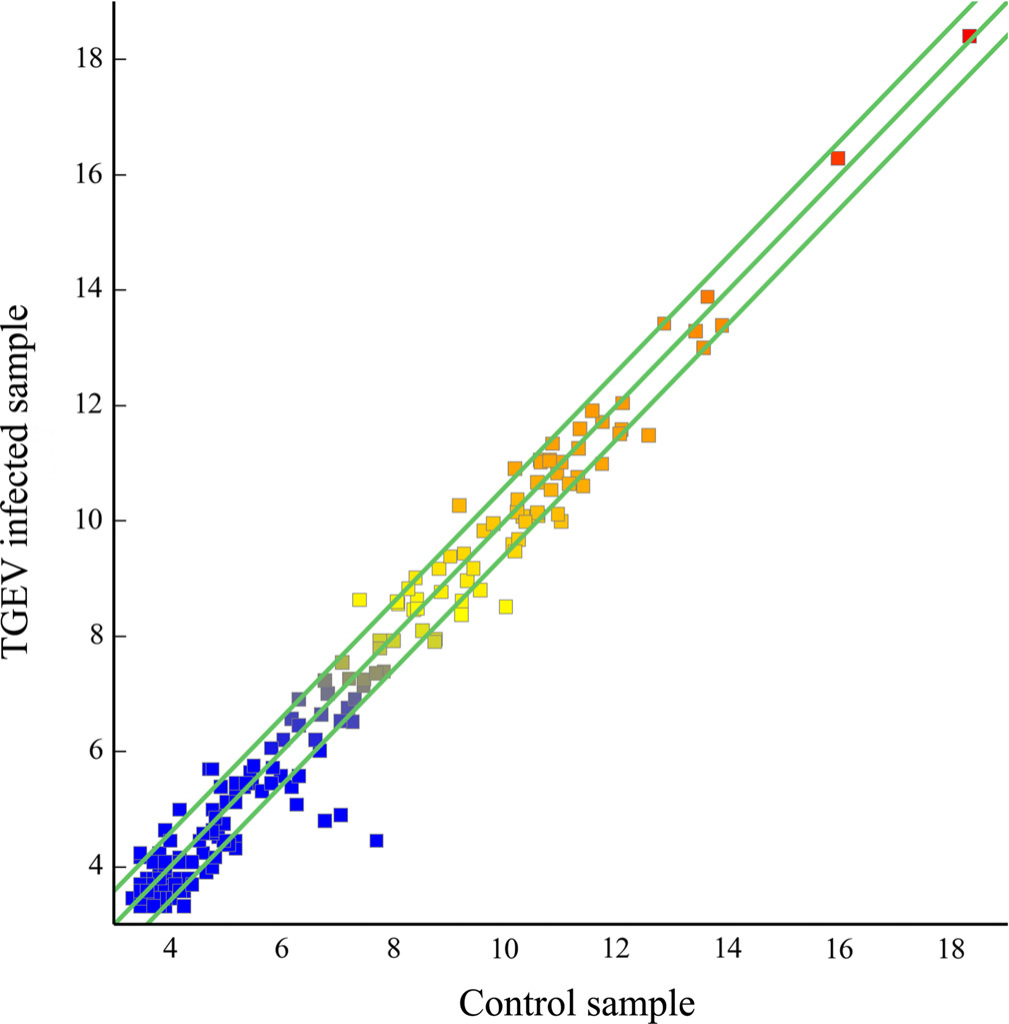
Scatter plot of the high-throughput sequencing data. The high-throughput sequencing data (differentially expressed miRNAs) are graphed on the scatter plot to visualize variations in miRNA expression between TGEV infected and control groups. The values on the X axes and Y axes of the scatter plot are the normalized values for the TGEV infected and control groups (log2 scaled). The green lines are fold-change lines (default fold-change value: 1.5).

Analysis of library sequencing data resulted in the identification of the 59 unique miRNAs that displayed significant differential expression between the infected ST cell and normal ST cell samples. Among these 59 miRNAs, 15 were significantly upregulated, while 44 were significantly downregulated upon TGEV infection (Fig. 3). The differentially expressed miRNAs data are graphed on the scatter plot (Fig. 4).

### Confirmation of differential expression of miRNAs

Stem-loop RT-qPCR detection assays were used to confirm the expression of certain novel miRNAs and differentially expressed miRNAs in normal ST cell and the infected ST cell samples. The selected miRNAs comprised four differentially expressed miRNAs (ssc-miR-19a, ssc-miR-28–3p, ssc-miR-92a and ssc-miR195) and four novel miRNAs (chr13–4473, chr6–12459, GL892871–2–16764 and chr9–15250). There were some differences in the results, because of the use of different technologies, but the results showed a general consistency between stem-loop RT-qPCR and high-throughput sequencing (Table 2).

**Table 2:** Stem-loop RT-qPCR confirmation for miRNAs.

Mature miRNA ID	Pre-miRNA ID	qRT-PCR fold-change (Infected/control)	High throughput sequencing fold-change (Infected/control)
ssc-miR-146b	ssc-mir-146b	+2.44	+3.30
ssc-miR-155–5p	ssc-mir-155	+1.35	+1.74
ssc-miR-30c-5p	ssc-mir-30c-1	+3.24	+2.29
ssc-miR-184	ssc-mir-184	+1.59	+2.15
ssc-miR-15b	ssc-mir-15b	-0.71	-0.50
ssc-miR-19a	ssc-mir-19a	-0.57	-0.47
ssc-miR-378	ssc-mir-378–1	-0.62	-0.58
ssc-miR-423–5p	ssc-mir-423	-0.83	-0.58
ssc-miR-140–3p	ssc-mir-140	-0.76	-0.38
ssc-miR-148a-3p	ssc-mir-148a	-0.59	-0.35

Four differentially expressed miRNAs and four novel miRNAs were selected from the high-throughput sequencing datasets and confirmed by stem-loop RT-qPCR. The results showed a general consistency between the stem-loop RT-qPCR and the high-throughput sequencing. The fold changes (miRNA copy numbers of TGEV-infected ST cell sample *vs*. miRNA copy numbers of normal ST cell sample) are shown in the diagram. The fold change cutoffs of the upregulated miRNAs and the downregulated miRNAs were 1.5 and 0.67, respectively. “+” and “–” indicate upregulated and downregulated miRNAs, respectively. qRT-PCR Ct threshold: 0.015.

### Target prediction and gene functional annotation of the targets of differentially expressed miRNAs

**Fig 5:**
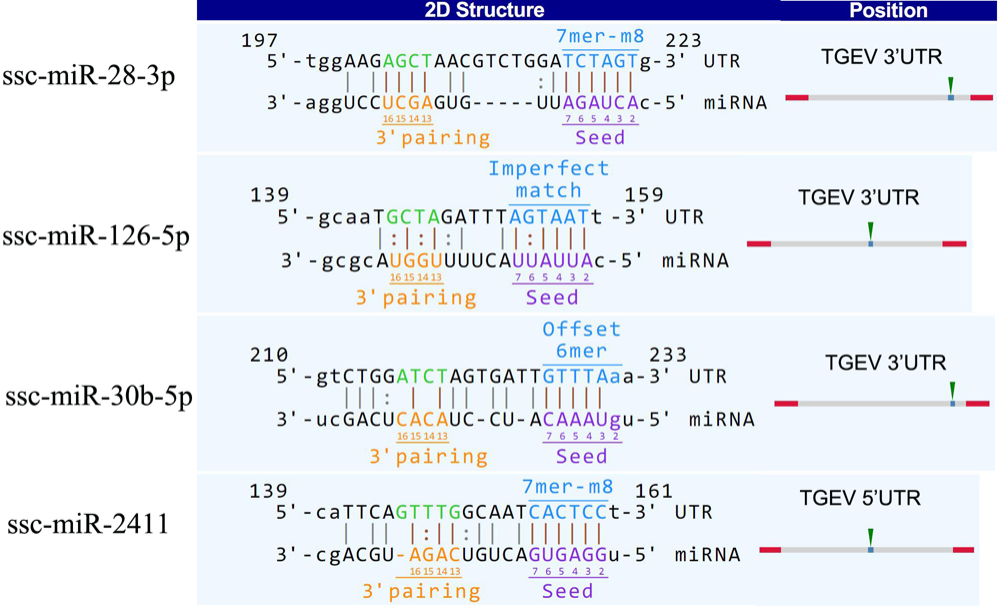
Differentially expressed miRNAs directly targeted the 3' UTR or 5' UTR of the TGEV genome. ssc-miR-28–3p, ssc-miR-126–5p and ssc-miR-30b-5p target the 3' UTR of the TGEV genome (28297 bp–28571 bp), ssc-miR-2411 target the 5' UTR of the TGEV genome (1 bp–303 bp).

The putative target genes of the 59 differentially expressed miRNAs (miRBase annotated) and 241 novel miRNAs were predicted using on-line miRNA target prediction tools to probe the biological roles of the miRNAs (S3 Table). To predict the putative TGEV targets for the differentially expressed miRNAs, the miRNA target gene database miRGen 3.0 was used with stringent criteria. The results showed that some of the differentially expressed miRNAs directly targeted the 3' UTR or 5' UTR of the TGEV genome: ssc-miR-28–3p, ssc-miR-126–5p and ssc-miR-30b-5p target the 3' UTR (28297 bp–28571 bp) and ssc-miR-2411 and chrX-16275 target the 5' UTR (1 bp–303 bp) (Fig. 5). This study also found a series of miRNAs that target host-encoded pre-miRNAs; for example, ssc-miR-9, ssc-miR-19a, ssc-miR-142–5p, ssc-miR-134 and ssc-miR-20c-5p all target ssc-mir-21. GO annotation was performed for the target genes of five designated differentially expressed miRNAs (miR-146b, miR-155–5p, miR-195, miR-124a and miR-1306–5p). The results showed that the differential expressed miRNAs and their target genes constituted a complex regulatory network: multiple miRNAs were linked through their common target genes. This complex regulatory network could regulate the expression of multiple genes through one miRNA, but could also regulate the expression of certain genes through several miRNAs in combination (S1 Fig. and S2 Fig.). For example, the significantly differentially expressed miRNAs, miR-9, miR-9–1 and miR-9–2 all target IL12A, but the immune-related gene IL7R was individually regulated by miR-140–3p. The in-depth analysis of the miRNA regulatory mechanism in gene expression will contribute to our understanding of the complexity of eukaryote genomes and gene regulatory networks.

**Fig 6:**
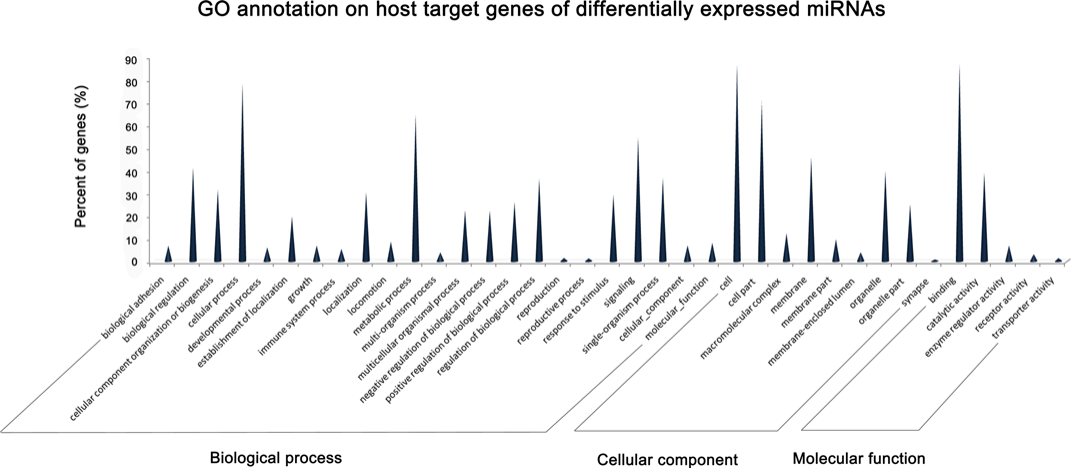
GO annotation of host target genes of differentially expressed miRNAs. GO functional analysis using the DAVID web-based tool shows that a series of targets belong to a series of functional genes involved in cellular process, metabolic process, and immune system process, which indicated the potential regulatory role of the differentially expressed miRNAs in viral infection. For other enriched GO terms, please see S4 Table.

The target genes were associated with cellular components, molecular functions and biological processes; the GO enrichment analysis revealed that the target genes were functionally enriched in immune system process, cellular process, metabolic process and others (Fig. 6; S4 Table).

KEGG analysis identified 143 overrepresented pathways, suggesting that these signaling pathways are regulated by the differentially expressed miRNAs during virus infection (S5 Table).

The analysis showed the target genes of the differential expressed miRNAs belong to the T cell receptor signaling pathway, which is involved in the regulation of T cell development, cytokine production and the activation of induced cell death, which is crucial for anti-virus activity. Pathways involved in cancer suggested that the differential expressed miRNAs play a regulatory role in cell proliferation and cell cycle progression.

## Discussion

TGEV is highly contagious. Once pigs are infected with the virus, it can spread to the entire breeding farm in 2 or 3 days. Dogs, cats and foxes can carry TGEV, but TGEV is not pathogenic for these animals. In addition, birds can spread TGEV, especially starlings. Similar to porcine epidemic diarrhea virus (PEDV), transmissible gastroenteritis virus of swine (TGEV) also belongs to the genus *Coronavirus*, family *Coronavirus*, *Nidovirales*. The clinical symptoms caused by the infection of these two viruses are quite similar, TGEV and PEDV infection have caused serious damage to the farming industry [20, 21]; however, compared with other *Coronavirus*, research on TGEV still lags behind. Currently, there are no commercial biological products for TGEV infection, and there is also no effective treatment for porcine transmissible gastroenteritis. Research on the molecular regulatory mechanisms acting during TGEV infection would have great significance for the prevention of infection by other *Coronaviruses*, including Human Coronavirus (HCoV) and Severe Acute Respiratory Syndrome Coronavirus (SARS-CoV).

Continuing miRNA research has indicated that miRNAs are involved in the interaction between a variety of viruses and their hosts. This phenomenon provided a paradigm shift in our understanding of latent infection and reactivation mechanisms of the virus. MiRNAs and their targets may represent new antiviral targets for therapy.

Recent studies indicate that host-encoded miRNAs play an important regulatory role in the virus life cycle. [22]. However, the host-encoded miRNAs do not always play an inhibitory role during viral infection. [23–27]. Host-encoded miRNA expression profiles can be changed by virus infection and virus-encoded miRNAs can also regulate the viral infection status and the expression of viral proteins through complementarity with the 3' UTR of the target gene. [28–32]

Previous studies showed that TGEV could effectively proliferate in ST cells; therefore, the ST cell line is an important culture system for TGEV research. The ST cell culture system can improve the immunogenicity of the vaccine relative to other cell lines; therefore, the ST cell line is also the ideal cell line for the preparation of a TGEV vaccine.

Currently, there are a very limited number of publications on miRNA expression profiles in porcine cells, and none that compare the differential expression profiles between passaged porcine testicular cells and primary porcine testicular cells. Studies on miRNA expression profiles after viral infection and the regulation mechanisms of miRNAs during viral infection are also in their initial stages.

In this study, TGEV-infected ST cells were chosen as the research subjects, and we preliminarily explored the miRNA molecular regulatory pathways during infection with the virus. We believe that the results of this study will assist with the prevention and treatment of the viral disease.

In a previous study of miRNA expression profile of the porcine reproductive system, 732 mature miRNAs were detected, but the study did not involve the miRNA expression profiles of a testicular cell line [33–34]. In this study, ST cell samples were collected after infection with TGEV, 241 novel host-encoded miRNAs were identified. MiRNAs frequently vary compared with their miRBase reference sequences. In this study, precursor miRNAs produced multiple mature variants (isomiRs). It appeared that much of the isomiR variability could be explained by variability in either Dicer or Drosha cleavage positions within the pre-miRNA hairpin.

MiRNAs have a regulatory impact on virus transcripts to support the viral replication cycle and allow the virus to escape the host immune system [35, 36]. We concluded that there is a very important relationship between these miRNAs and the replication or translation of TGEV. Further study of the mechanism is essential for virus prevention or treatment.

The immune system is the crucial barrier for resisting viral infection. In recent years, research on molecular regulation mechanism of mammalian immune-related genes has made tremendous progress, but the details of the molecular regulation mechanism of immune-related genes that are mediated by miRNA remain unknown.

In recent years, the role of miRNAs in the immune response has been revealed. Evidence shows that the mammalian immune response is associated with the regulation of miRNA. Recent research showed that the pathogen-associated molecular pattern (PAMP), pattern receptors (PRR) induced miRNA expression via a series of signaling pathways, thereby regulating innate immunity. Therefore, the identification and characterization of the regulatory miRNAs involved in the innate immune response is essential for the in-depth study of animal immune regulatory mechanisms and the treatment of the viral diseases [37].

In this study, a large number of immune-related miRNAs were significantly differentially expressed upon infection by TGEV. In particular, mature miR-146 and miR-155 were significantly upregulated in infected ST cells compared with the control group; the fold changes of miR-146 and miR-155 between the infected ST cells and the control group were 3.30 and 1.74, respectively. Previous research showed that miR-146 and miR-155 play a key regulatory role on host immune function.[38–40]. In this study, the sequencing data confirmed the results of previous studies, and proved that in addition to immune cells, normal cells also abundantly expressed miR-146 and miR-155 when stimulated by viral antigens and are involved in the regulation of immunity.

## Materials and Methods

### Virus and cells

TGEV Sichuan strain and the ST cell line (Provided by Animal Biotechnology Center of Sichuan Agricultural University) were used in this study. Cell cultures were performed at 10^6^ cells/175 cm^2^ dishes and were maintained under standard conditions of 37°C and 5% CO_2_ in modified RPMI-1640 nutrient solution (Thermo Fisher Scientific, Waltham, UK), supplemented with 10% heat-inactivated fetal bovine serum and 50 mg/ml penicillin/streptomycin antibiotic solution (GIBCO, Beijing, China).

The experiment consisted of pooled samples, samples were performed in triplicates. When the cells were 70% confluent in the culture dishes, they were infected with TGEV at 10 PFU per cell. The infected and control cells groups were harvested at 72 post infection (hpi), and resuspended in Trizol (Invitrogen, Shanghai, China) and stored at-70°C. All infected and control samples were combined into a single control group and a single infected group, respectively.

### Data sources

The full genomic sequence of TGEV has been determined and is available in the GenBank Genome database (ftp://ftp.ncbi.nih.gov/refseq/release/viral/) (accession no.: DQ811788). The Sus scrofa genomic sequence and gene annotation information are available at the University of California Santa Cruz (UCSC) Genome browser (http://genome.ucsc.edu/index.html). Novel annotated pig-encoded miRNAs are available in miRBase (http://www.mirbase.org/).

### RNA isolation

The total RNA was extracted from the TGEV-infected ST cells and control ST cells using the Trizol reagent (Invitrogen), according to the manufacturer’s instruction. The NanoDrop ND-1000 spectrophotometer (Nano Drop Inc., Wilmington, DE, USA) was used to accurately measure the concentration (OD 260) and protein contamination (Ratio OD260/OD280) of the total RNA samples. The Agilent 2100 Bioanalyzer (Agilent, Beijing, China) was used to accurately assess the quality and concentration of the sequencing library. Denaturing agarose gel electrophoresis was used to assess the RNA integrity and gDNA contamination. The same RNA samples were used in the stem-loop RT-PCR experiments to verify the expression of specific miRNAs. Total RNA of infected and control samples were mixed during high-throughput sequencing, respectively.

### Small RNA library construction and sequencing

Total RNA of each sample was used to prepare the miRNA sequencing library, which included the following steps: 1) 3'-adapter ligation with T4 RNA ligase 2 (truncated); 2) 5'-adapter ligation with T4 RNA ligase; 3) cDNA synthesis with an RT primer; 4) PCR amplification; and 5) extraction and purification of 120–140 bp PCR amplified fragments (corresponding to ~15–25 nt small RNAs) from polyacrylamide gels. An Agilent 2100 Bioanalyzer quantified the libraries, after which the samples were diluted to a final concentration of 8 pM and cluster generation was performed on the Illumina cBot using TruSeq Rapid SR cluster kit (#GD-402–4001, Illumina), following the manufacturer’s instructions. The DNA fragments in the libraries were denatured with 0.1M NaOH to generate single-stranded DNA molecules, captured on Illumina flow cells, amplified *in situ* and finally sequenced for 36 cycles on Illumina HiSeq 2000 (Illumina, San Diego, CA, USA), according to the manufacturer’s instructions [41].

### miRNA-seq data analysis

After the sequencing images were generated, image analysis and base calling were performed using Off-Line Basecaller software (OLB V1.8.0) (http://support.illumina.com/sequencing/sequencing_software/offline_basecaller_olb.ilmn). Subsequently, 3' adapter sequences were trimmed from clean reads (reads that passed Solexa CHASTITY quality filter) and the reads shorter than 15 nt were discarded. Known sequences of mRNA, rRNA, tRNA, snRNA, and snoRNA were also excluded. The 3'-adapter-trimmed-reads (≥ 15 nt) were aligned to the latest known *Sus scrofa* reference miRNA precursor set in the Sanger miRBase 19 (http://www.mirbase.org/), using Novoalign (v2.07.11). Reads with counts < 2 were discarded when calculating the miRNA expression. To characterize the isomiR variability, any sequence that matched the miRNA precursors in the mature miRNA region ±4nt (no more than one mismatch) were accepted as mature miRNA isomiRs, which were then grouped according to the 5-prime (5p) or 3-prime (3p) arm of the precursor hairpin. miRDeep2 (http://www.mdc-berlin.de/en/research/research_teams/systems_biology_of_gene_regulatory_elements/projects/miRDeep/) was used to predict novel pig-encoded miRNAs. For novel miRNA prediction, we pooled all sequence data from the 3' adapter-trimmed files and all adapter-trimmed sequences of length <17 bp and with >1 mismatch were excluded from prediction pipeline. Mfold software (http://mfold.rna.albany.edu/) and the Vienna RNAfold web server (http://rna.tbi.univie.at/cgi-bin/RNAfold.cgi) predicted the fold-back secondary structures of the novel miRNAs, under default folding conditions. The ratio of the normalized read counts between two samples based on the most abundant isomiR read counts.

The IDEG6 web tool (http://telethon.bio.unipd.it/bioinfo/IDEG6/) was used to determine the significance of the observed differences in miRNA counts between the two samples. When comparing differential miRNA expression between two samples (TGEV infected ST cells/ Control ST cells), differentially expressed miRNAs may be determined by fold change filtering, normalized most abundant tag counts were used for fold-change calculation. In the present study, the fold change ≥ 1.5 indicates that the miRNA in the TGEV-infected sample is up-regulated, while a fold change ≤ 0.67 indicates that the miRNA in a PCMV-infected sample is down-regulated (We identified differentially expressed miRNAs based on the normalized most abundant tag counts).

### Target prediction and functional enrichment of differentially expressed miRNAs

The miRNA target gene database miRGen 3.0 (http://www.diana.pcbi.upenn.edu/miRGen.html) was used to predict miRNA target genes and for bioinformatic analysis of gene function. This database integrates four major miRNA target prediction tools: PicTar (http://pictar.mdc-berlin.de/), miRnada (http://www.microrna.org/microrna/home.do), DIANA-microT (http://diana.cslab.ece.ntua.gr/microT/) and TargetScanS (http://genes.mit.edu/tscan/targetscanS2005.html) [42–46].

The Gene Ontology (GO) project provides a controlled vocabulary to describe gene and gene product attributes in any organism (http://www.geneontology.org). The ontology covers three domains: Biological Process, Cellular Component and Molecular Function. The target genes of differentially expressed miRNAs were subjected to GO analysis on the web-based tool Database for Annotation, Visualization, and Integrated Discovery (DAVID, http://david.abcc.ncifcrf.gov/). The significance of the gene function screening of the corresponding target genes was performed at a P-value ≤0.05.

Pathway analysis is a functional analysis that maps genes to the Kyoto Encyclopedia of Genes and Genomes (KEGG) pathways. The P-value (EASE-score, Fisher-Pvalue or Hypergeometric-Pvalue) denotes the significance of the pathway correlated to the conditions. The lower the P-value, the more significant the pathway (the recommended P-value cut-off is 0.05).

### Stem-loop RT-PCR analysis

Stem-loop RT-PCR was carried out using the SYBR Green PCR Core Reagents Kit (Applied Biosystems, Foster City, CA, USA), according to the manufacturer’s instructions. The transcription product, miRNA-specific forward primer and universal reverse primer were used in the stem-loop RT-PCR test to confirm the expressions of miRNAs (S6 Table). The amplification conditions were: 94°C 3 min; 40 cycles of 95°C 20 s, 60°C 40 s and 72°C 20 s; and elongation at 72°C for 5 min. Stem-loop RT-PCR reactions were performed using the ABI Prism 7900 Sequence Detection System (Applied Biosystems), according to the manufacturer’s instructions [47, 48].

### Conclusions

This is the first comprehensive study of the miRNA expression profile of ST cells after infection with TGEV using a deep sequencing approach. 241 pig-encoded novel miRNAs were identified. We also analyzed the miRNA regulatory network, identified differential expressed miRNAs between the ST cell line and TEGV-infected ST cells, and performed GO analysis for target genes of the differentially expressed miRNAs. We believe that the data and the analysis will contribute to the understanding of miRNA regulation mechanisms during viral infection, and will lead to new approaches for the treatment or prevention of viral diseases.

## Supporting Information

S1 Fig A predicted network between the 15 upregulated miRNAs and their putative target genes in the pig genome. (PDF)

S2 Fig A predicted network between the 44 downregulated miRNAs and their putative target genes in the pig genome. (PDF)

S1 Table Expression profile of known detected porcine miRNAs. (XLS)

S2 Table Novel porcine miRNAs expressed in ST cells. (XLS)

S3 Table Differentially expressed miRNAs. (XLS)

S4 Table GO functional enrichment annotations for the miRNA targets. (XLS)

S5 Table KEGG pathway annotations for the miRNAs targets. (XLS)

S6 Table Primers used for stem-loop RT-PCR. (XLSX)
