# Supplementary figures and images for: The Porcine MicroRNA Transcriptome Response to Transmissible Gastroenteritis Virus Infection
Source: PLoS One. 2015 Mar 17;10(3):e0120377. doi: 10.1371/journal.pone.0120377 (PMC4363316; doi:10.1371/journal.pone.0120377)

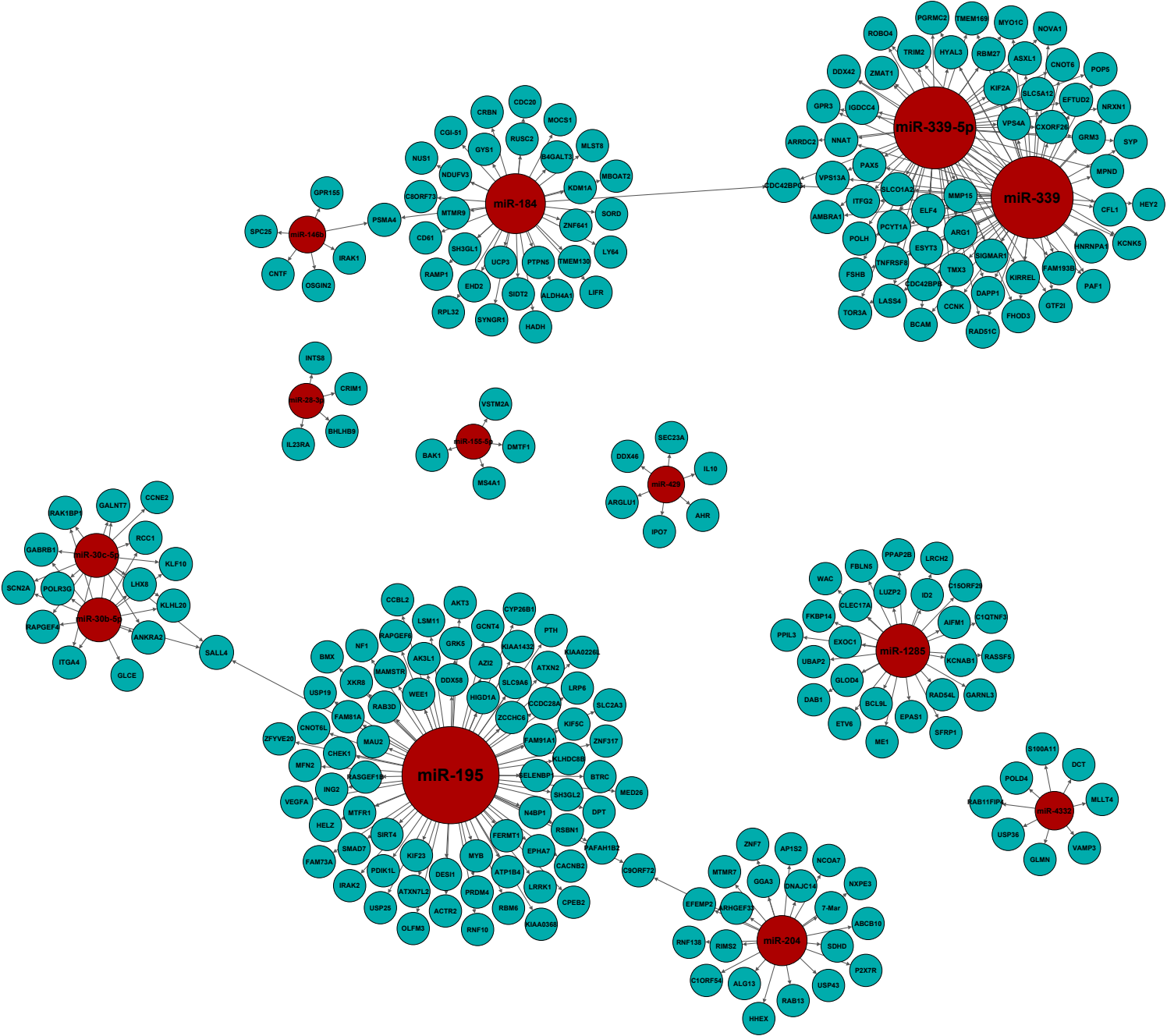

Supplement: S1 Fig — (PDF) [file pone.0120377.s001.pdf]

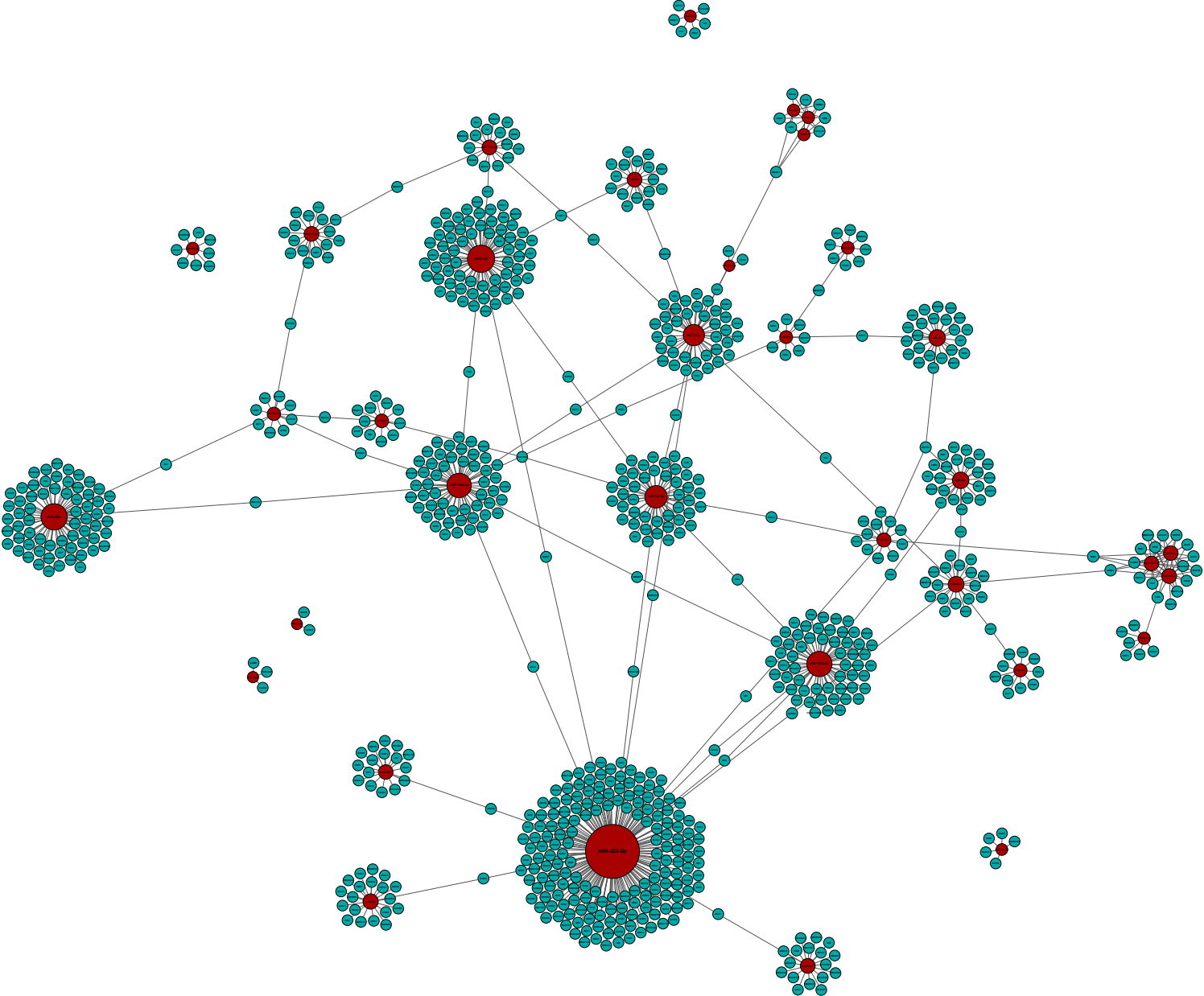

Supplement: S2 Fig — (PDF) [file pone.0120377.s002.pdf]
